# Ultra-rapid detection of SARS-CoV-2 in public workspace environments

**DOI:** 10.1371/journal.pone.0240524

**Published:** 2021-02-24

**Authors:** Ozlem Yaren, Jacquelyn McCarter, Nikhil Phadke, Kevin M. Bradley, Benjamin Overton, Zunyi Yang, Shatakshi Ranade, Kunal Patil, Rishikesh Bangale, Steven A. Benner

**Affiliations:** 1 Foundation for Applied Molecular Evolution, Alachua, Florida, United States of America; 2 GenePath Diagnostics Inc., Ann Arbor, Michigan, United States of America; 3 GenePath Diagnostics India Pvt. Ltd., Pune, Maharashtra, India; 4 Firebird Biomolecular Sciences LLC, Alachua, Florida, United States of America; University of Helsinki, FINLAND

## Abstract

Managing the pandemic caused by SARS-CoV-2 requires new capabilities in testing, including the possibility of identifying, in minutes, infected individuals as they enter spaces where they must congregate in a functioning society, including workspaces, schools, points of entry, and commercial business establishments. Here, the only useful tests (a) require no sample transport, (b) require minimal sample manipulation, (c) can be performed by unlicensed individuals, (d) return results on the spot in much less than one hour, and (e) cost no more than a few dollars. The sensitivity need not be as high as normally required by the FDA for screening asymptomatic carriers (as few as 10 virions per sample), as these viral loads are almost certainly not high enough for an individual to present a risk for forward infection. This allows tests specifically useful for this pandemic to trade-off unneeded sensitivity for necessary speed, simplicity, and frugality. In some studies, it was shown that viral load that creates forward-infection risk may exceed 10^5^ virions per milliliter, easily within the sensitivity of an RNA amplification architecture, but unattainable by antibody-based architectures that simply target viral antigens. Here, we describe such a test based on a displaceable probe loop amplification architecture.

## Introduction

Once again, the world is facing a coronavirus pandemic, this time from SARS-CoV-2 that emerged in Wuhan, China in late 2019 [1]. Previous coronaviral threats include the SARS coronavirus that emerged in southern China in 2003 as the causative pathogen of severe acute respiratory syndrome [2, 3], and a coronavirus that causes Middle East respiratory syndrome (MERS) [4–6]. However, unlike these homologous coronaviruses, SARS-CoV-2 creates a remarkable range of medical outcomes, from lethality to mild (or no) symptoms in infected individuals [7, 8]. Further, it appears to be transmissible via asymptomatic carriers. This, in turn, has caused economic disruption across the globe that is measured in the trillions of dollars [9].

At present, no single event seems likely to resolve this pandemic cleanly. Coronaviruses have generally been poor targets for vaccines [10, 11], although novel routes may make this view obsolete [12]. Antivirals that are effective against other viruses [13] may have activity against SARS-CoV-2 [14], but their ability to manage the pandemic remain in doubt. Treatments that mitigate disease symptoms may save lives, but are not likely to be effective at preventing virus spread [15, 16].

All of these factors create an urgency for tests that identify, in minutes, low- or asymptomatic infected individuals as they enter public spaces, such as workspaces, schools, points of entry, and commercial business establishments. To be useful, such tests must (a) require no sample transport, (b) require minimal sample manipulation, (c) can be performed by unlicensed individuals, (d) return results on the spot in much less than one hour, and (e) cost no more than a few dollars. They cannot involve RNA extraction or other sample preparation steps found in assays typically used in reference laboratories.

Such specs are demanding. However, the demands are mitigated by the fact that to meet its societal purpose, the test need not be ultra-sensitive. To identify carriers who have the potential to infect individuals in public spaces, tests need only be sensitive enough to catch perhaps 10^4^−10^5^ virions per sample from the nasal or oral cavities. While these numbers remain to be better defined (and on-site tests are likely to help to define them), it is clear that the necessary speed, simplicity, and frugality can be more easily obtained by trading off unneeded sensitivity. False positives are managed by re-testing. While false negatives remain (either by failure of low resource sampling or failure of the test itself), the absence of a work-place test effectively makes all untested low- or asymptomatic carries "false negatives".

Using various nucleic acid based architectural innovations, we have developed a variety of kits that meet these specs with untrained and unlicensed users outside of traditional laboratory settings, including in the field [17–19]. These have focused on detecting environmental pathogens, including detecting arboviral RNA in infected insects and ticks [20] since these require no regulatory supervision. We have established many collaborations, including TrakItNow (which builds mosquito traps) [21], immediate care facilities [22], LynxDx in Ann Arbor, MI, and Achira Labs in Bangalore, India [23].

Here, we report the application of these architectures to the detection of the SARS-CoV-2 coronavirus in nasal and oral cavity samples. The architectures that we report here:

- Operate on dry swabbed samples, without extensive sample preparation,
- Require no temperature cycling, and do not require expensive instruments,
- Have ~$3.00 in disposable costs, and therefore are routinely usable,
- Produce an easily read signal in less than 30 minutes,
- Have limits of detection of ~ 200 viral RNA per assay when minimal sample preparation was sought, and
- Are easy to run, not requiring a trained medical professional.

## Material and methods

### Primers and displaceable probes for displaceable probe RT-LAMP (DP-RT-LAMP)

RT-LAMP primers were designed as described previously [17, 18]. The DP-RT-LAMP primers and strand displaceable probes were purchased from Integrated DNA Technologies (IDT, Coralville, IA). Strand-displaceable probes were 5’-labeled with Iowa Black-FQ (IBFQ) and 3’-labeled with FAM. For multiplexed LAMP, internal control probes targeting the human RNase P gene were 5’-labeled with IBFQ and 3’-labeled with JOE. The duplex segment of the displaceable probes was screened against viral and human genomic overlaps (see **S1 Table** in **S**1 **File**).

### DP-RT-LAMP assay

12.5 μL of 2X WarmStart LAMP master mix (NEB) was combined with 2.5 μL of 10X LAMP primer set, 1 μL of excess B3 primer (300 μM), 0.2 μL of dUTP (100 mM, Promega), 0.5 μL of Antarctic Thermolabile UDG (1U/μL, NEB), 0.5 μL of RNase inhibitor (40U/μL, NEB), and 8 μL of sample (consisting of either heat-inactivated virus or nasal/saliva samples spiked with heat-inactivated virus). No template control used nuclease-free water to replace the sample.

10X LAMP primer set consists of 16 μM each of FIP and BIP, 2 μM each of F3 and B3, 5 μM LF (or LB for CoV2-v2-4 set), 4 μM LB (or LF for CoV2-v2-4 set), 150 nM quencher-bearing probe, and 100 nM of fluorophore-bearing probe.

Reactions were monitored in real-time using either a LightCycler^®^ 480 (Roche Life Science, US) or a Genie^®^ II (Optigene, UK) instrument. 8-strip PCR tubes were first incubated at 55°C for 10 min followed by incubation at 65°C for 45–60 min. During the 65°C incubation, fluorescence signal was recorded every 30 seconds using FAM/SYBR channel of the instrument.

End-point observation of the fluorescence signal was enabled by blue LED light (excitation at 470 nm) through orange filter of SafeBlue Illuminator/ Electrophoresis System, MBE-150-PLUS (Major Science, US) or 3D printed observation box (Firebird Biomolecular Sciences, US).

### Sample preparation

#### Heat-inactivated SARS-CoV-2 isolate

Authentic SARS-CoV-2, isolate USA-WA 1/2020, was obtained through BEI Resources (cat no. NR-52286, 1.16×10^9^ genome equivalents/mL). This virus has been inactivated by heating at 65°C for 30 minutes. Target dilutions were made in 1 mM Na citrate pH 6.5, 0.4 U/μL RNase inhibitor (NEB, Ipswich, MA) and aliquots (100 μL) were stored at -80°C. This material was used for LOD experiments and spiking nasal swabs and saliva samples to create a positive control.

#### Nasal swab testing

CleanWIPE Swab, 3” Semi-Flexible bulb tip (HT1802-500, Foamtec International) was used for nasal sampling. Each nostril was swabbed for at least 10 seconds using the same swab. Swab was placed in sterile 15 mL falcon tube and stored at 4°C until processing. Swabs were processed within 1 hour.

Nasal swabs were eluted in 200 μL of buffer solution (1 mM Na citrate pH 6.5, 2.5 mM TCEP, 1 mM EDTA, 10 mM LiCl, 15% Chelex-100) by brief vortexing. Swabs were then removed and elution solution was briefly spun down. An aliquot of the elution was added to DP-RT-LAMP assay.

#### Saliva samples

About 1 mL of saliva was collected in sterile 5 mL falcon tube and stored at 4°C until processing and samples were processed within 1h. A suspension (100 μL) of 15% Chelex-100 in 1.6 mL microcentrifuge tube was spun down briefly and supernatant was removed. To this was added 100 μL of saliva mixed with 1 μL of concentrated sample preparation solution (0.1 M Na citrate pH 6.5, 1M LiCl, 0.25 M TCEP, 0.1 M EDTA). Each sample was briefly vortexed and spun down to settle the Chelex-100. An aliquot of the sample was added to DP-RT-LAMP assay.

*Collecting saliva on Q-paper*. Q-paper was first dipped into saliva samples and soaked for 5 seconds, then air dried for 5 min. Saliva soaked Q-paper was directly inserted into 50 μL of DP-RT-LAMP mixture (see **S**1 **File** for Q-paper preparation).

#### Evaluation of clinical samples

Nasopharyngeal (NP) swabs, previously stored in VTM media, were eluted in 200 μL buffer solution (1 mM Na citrate pH 6.5, 2.5 mM TCEP, 1 mM EDTA, 10 mM LiCl, 15% Chelex-100) by brief vortexing. Swabs were then removed and elution solution was briefly spun down. 5 μL of sample was added to DP-RT-LAMP assay.

Reactions were monitored in real-time using Rotor Gene Q (Qiagen, US). FAM and JOE channels were used for SARS-CoV-2 and RNase P detection, respectively.

#### Ethical statement

*Nasal and Saliva samples*. For contrived samples, nasal and saliva samples were collected from healthy volunteers and spiked with heat-inactivated virus. Samples were collected according to IntegReview IRB procedure (protocol number: 2020001). Study was advertised through written communication and samples were accepted from adult volunteers (18 years old or older) during the month of September in 2020. Since it is not a clinical evaluation of active infection, study group consisted of only adults. Each participant was provided with information sheet explaining the details of the study along with the consent form to sign. Participant signed the form in the presence of a witness at the start of the study and a copy of the informed consent form has been provided to the participant.

All volunteers were screened for fever immediately prior to donating samples. Criteria that would exclude potential volunteers from participating include fever and signs of illness not within the nature of seasonal allergies (that is, respiratory congestion in combination with a fever). Exclusion from one round of sample collection will not disqualify a volunteer from future participation or sample collection. Upon each round of collections, the inclusion/exclusion criteria evaluation will be performed, and any volunteers that qualify may participate, even if they have been excluded from previous collections. Participants contributed to this study may not be considered representative of a larger population. Table of relevant demographic details are provided below. Collected samples were then spiked in with heat-inactivated SARS-CoV-2 virus at varying concentrations and remaining of the samples were disposed in a biohazard bag containing 10% bleach at the end of the experiment.

**Table:** 

**IRB procedure 2020001**					
**Gender**	**Race**	**Age**	**Gender**	**Race**	**Age**
Male	Caucasian	25	Male	Asian	46
Male	Hispanic	44	Female	Caucasian	40
Male	Caucasian	43	Female	Caucasian	33
Male	Asian	51	Female	Hispanic	44
Male	Caucasian	51	Female	Caucasian	65
Male	Asian	45	Female	Caucasian	38
Male	Caucasian	27	Female	Hispanic	27

In addition to IRB approved saliva collection, some of the saliva samples were purchased from BioIVT (human saliva, 5 mL) and received as de-identified. Donor information sheet with gender, age and race was provided (see table below).

**Table:** 

**BioIVT human saliva**					
**Gender**	**Race**	**Age**	**Gender**	**Race**	**Age**
Male	Caucasian	35	Female	Caucasian	49
Male	Asian	29	Female	Hispanic	30
Male	Caucasian	31	Female	Caucasian	38
Male	Asian	28	Female	Caucasian	31
Male	Caucasian	33	Female	Hispanic	29
Male	Caucasian	27	Female	Hispanic	31
Male	Caucasian	30	Female	Caucasian	28
Male	Caucasian	35	Female	Asian	25
Male	Caucasian	27	Female	Caucasian	24
Female	Hispanic	26	Female	Hispanic	22

*Clinical nasopharyngeal (NP) samples collected by GenePath Dx*. Samples were collected and tested under a protocol reviewed and approved by GenePath Dx / Causeway Healthcare’s Independent Ethics Committee/ Institutional Review Board which is registered with the Central Drugs Standard Control Organization (CDSCO), Office of Drugs Controller General (DCG), Directorate General of Health Services, Ministry of Health and Family Welfare (MoHFW), Government of India (with registration number ECR/225/Indt/MH/2015). Nasopharyngeal and oropharyngeal swabs for routine Covid-19 testing were collected either at a drive-through facility or during a home visit. The demographics included individuals of all ages who were referred for Covid-19 testing by their physician on account of symptoms or potential exposure, or individuals who requested self-testing on account of symptoms, exposure or need for a test result for travel or other similar purposes. Individuals availing of these facilities were explained about the potential for use of leftover samples for test improvement, new test development, and statistical analysis in a manner where no personally identifiable information would be revealed. All individuals were informed that this work had been approved by a government approved ethics committee and no information from these tests would be communicated back to them. Verbal consent was obtained from individuals, who expressed interest in participating in this work, prior to sample collection. Samples collected between June–September 2020 were used in this work. The tubes of individuals who consented to such use of their samples were marked with an additional symbol. Consent was witnessed by the second collector from the collection team. Only samples from individuals who could directly give consent (i.e. no minors) were collected. There were no other inclusion or exclusion criteria. The samples may be considered as a representative of the larger population in the geographical area that they were collected in but cannot be considered a representation of a larger global population. Within the testing laboratory, samples are only marked with codes that cannot be decoded by the testing laboratory. Matching of samples by the reference method and novel method is only made through the codes and no individual names and IDs are available.

**Table:** 

**GenePath Dx samples from India**			
**Gender**	**Age**	**Gender**	**Age**
Female	26	Female	75
Male	34	Female	30
Female	29	Male	41
Female	53	Male	32
Male	79	Male	41
Male	26		

### Multiplexed DP-RT-LAMP to detect SARS-CoV-2 and RNase P (internal control)

For simultaneous detection of virus and internal control, total amount of LAMP primers was kept the same and half the amounts of primer mix was used for virus and internal control detection. For proof of concept, varying amounts of heat-inactivated virus was first tested in the presence of human RNA (440 copies).

Nasal and saliva samples were treated with an additional heat step (95°C for 5 min) after spiked with inactivated virus and treated with buffers described in sample preparation section. Multiplexed LAMP was analyzed on LightCycler^®^ 480 (Roche Life Science, US) using FAM channel (483–533) for SARS-CoV-2 detection and JOE channel (558–610) for RNase P detection.

## Results

### Assay architecture

In recent years, reverse transcription loop-mediated isothermal amplification (RT-LAMP) has become an alternative to RT-PCR due to its high sensitivity and specificity, its tolerance for inhibitory substances, and operation at constant temperatures. Together, these lower assay complexity and cost, making LAMP often considered for COVID-19 diagnostics [24–26].

In its classical form, RT-LAMP uses six primers binding eight distinct regions within a target RNA. It runs at constant temperatures ranging from 62°C to 72°C, and uses a reverse transcriptase and a DNA polymerase with strong strand displacing activity (e.g. Bst DNA polymerase). Initially, forward and backward internal primers (FIP and BIP), with outer forward and backward primers (F3 and B3), form a double loop structure. This structure facilitates exponential amplification by formation of multiple repeating loops and improvement in amplification time is usually obtained by additional loop primers (LB and LF) [27].

Classical LAMP generates signals by the precipitation of the magnesium salt as one of its byproducts, pyrophosphate; the turbidity from this precipitation is detected. Alternatively formation of high molecular weight amplicons allows an intercalating dye to create a fluorescent signal [28, 29] or non-fluorescent signal [30, 31]. Alternatively, the pH change arising during the amplification is detected by the change in the color of an indicator [32–34].

None of these are well suited for workplace detection of pathogen RNA, such as that from SARS-CoV-2. These detection architectures can easily be deceived by off-target amplicons, and are therefore susceptible to generation of false-positive results. Confirming the nature of the amplicon by measuring melting temperatures, very useful in PCR, is difficult with LAMP amplicons whose lengths mature over the time of the process, and where off-target amplicons have unpredictable melting temperatures [35–37]. Assimilating probes have been introduced to allow the high molecular weight amplicon to contain a fluorophore, where the assimilation separates the fluorophore from a fluorescence quencher [38].

**Fig 1:**
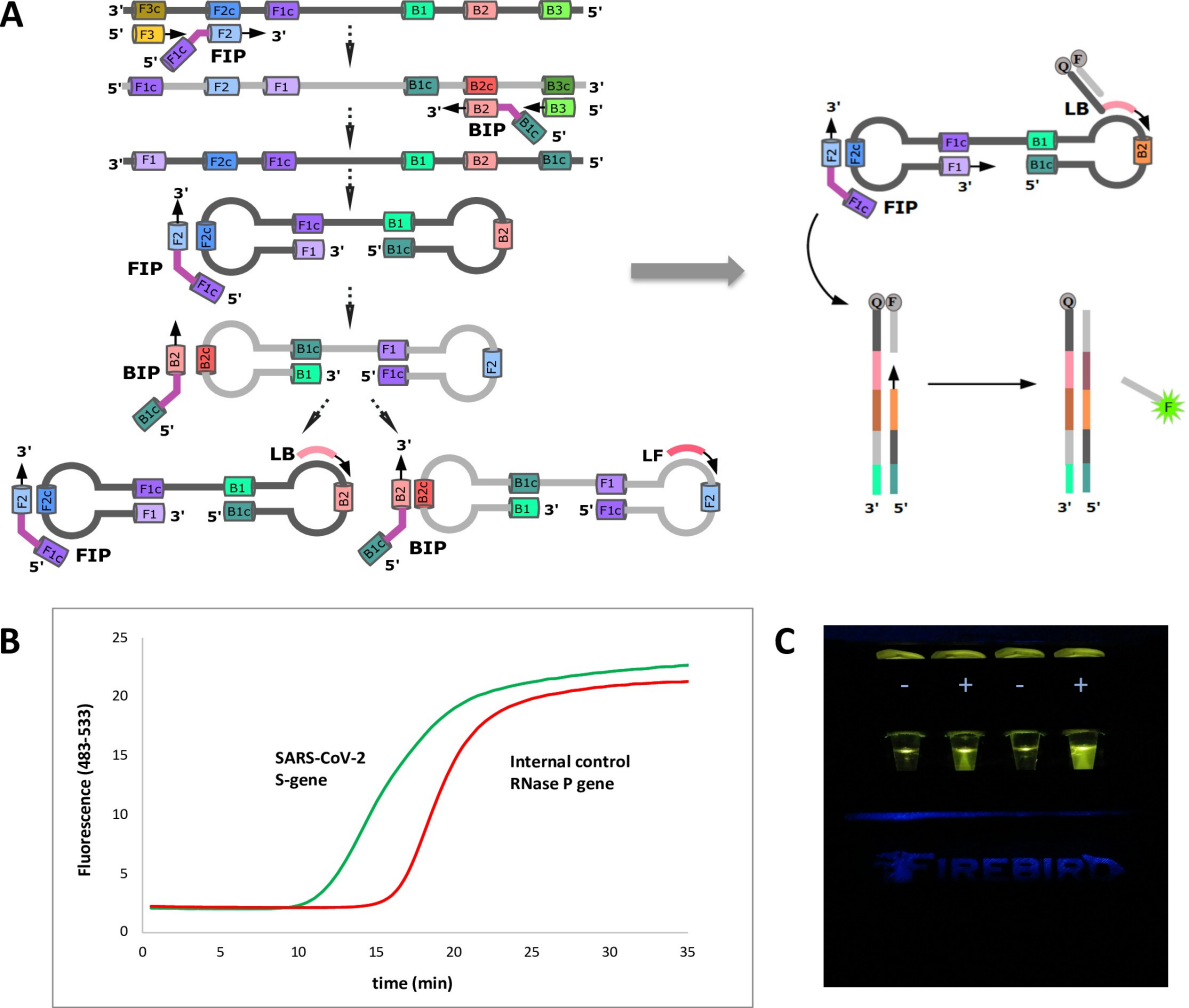
Displaceable probe LAMP to detect SARS-CoV-2. **(A)** Classical RT-LAMP utilizes six primers hybridizing to eight regions within the viral genome. These primers form a dumbbell structure through self-hybridization of FIP and BIP and addition of two loop primers improves the amplification rate. To allow simultaneous detection of several targets in real-time, displaceable probe architecture was employed by tagging one of the loop primers with a quencher and supplementing with a partially complementary probe containing a fluorophore tag. **(B)** Positive results can be analyzed in real-time and process manifests itself as sigmoidal curve as it would be in RT-qPCR using TaqMan probes. **(C)** End-point fluorescence is then observed through orange filter coupled with blue LED (exc. 470 nm, Firebird Biomolecular Sciences LLC, US).

To manage these issues, we offer an alternative architecture that exploits a displaceable probe (DP). This is a short oligonucleotide carrying a 3’-fluorophore that is displaced from a complementary oligonucleotide as the desired amplification proceeds. Complementary oligonucleotide has a 5’-quencher, and carries a tag that is a primer that binds to one of the loops in the LAMP double loop structure (**Fig 1A**). Thus, each probe is delivered to the amplification mixture as a target-sequence-independent double-strand probe region and a single-stranded target-priming region. This architecture allows to measure the fluorescent signal in real-time (**Fig 1B**). Alternatively, end-point visualization of the fluorescent signal can be realized through an orange filter by exciting the fluorophore with blue LED (**Fig 1C**).

### DP-RT-LAMP assays using heat-inactivated virus isolate

**Fig 2:**
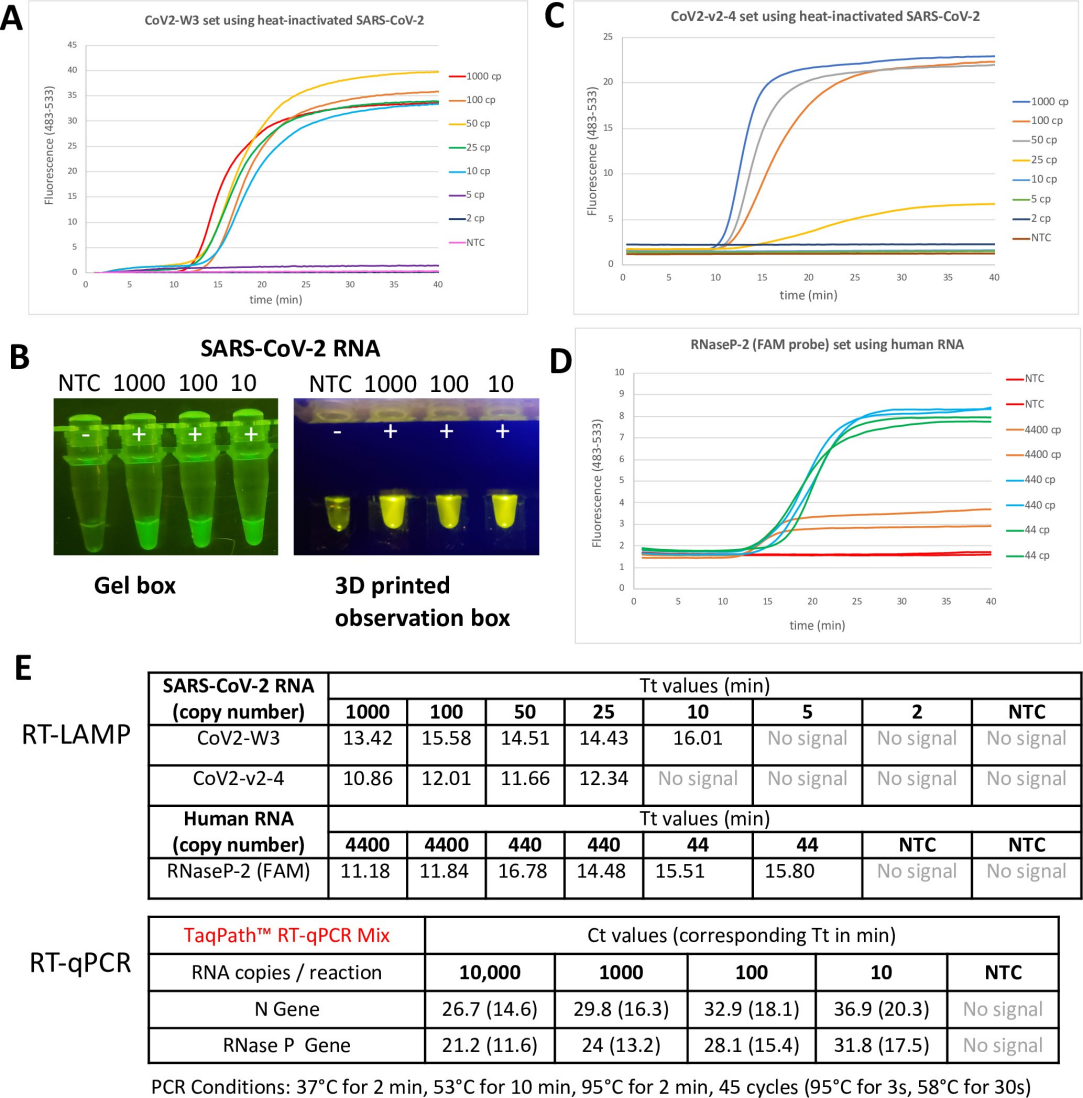
Limit of detection using DP-RT-LAMP primers using heat-inactivated SARS-COV-2 or human RNA (for internal control). **(A)** Real-time analysis of CoV2-W3 primer set (targeting S gene) with LOD of 10 RNA copies/assay. **(B)** End-point visualization of LAMP products with primer set CoV2-W3. **(C)** Real-time analysis of CoV2-v2-4 primer set (targeting N gene) with LOD of 25 RNA copies/assay. **(D)** Real-time analysis of internal control RNaseP-2 primer set (targeting human RNase P gene) with LOD of 44 copies of human RNA/assay. **(E)** Time to threshold (Tt) values of each LAMP primer set was determined for each target copy number/assay and similar values were obtained when compared to RT-qPCR test (Ct values were also converted to their corresponding Tt values for convenience).

We assessed the LOD with heat-inactivated SARS-CoV-2 isolate after running initial test with synthetic RNA (**S1 Fig** in **S**1 **File**). This was also used to "spike" nasal swabs and saliva samples. Among three conditions tested (**S2 Fig** in **S**1 **File**), the best sensitivity was achieved with using WS-RTx and excess B3 primer with incubation at 55°C for 10 min followed by incubation at 65°C 50 min. This protocol was used to assess the sensitivity of another SARS-CoV-2 primer set targeting N gene and internal control primer set targeting RNase P. The primer set targeting S gene (CoV2-W3) gave an LOD of 10 copies/assay within 16 min (**Fig 2A**); the fluorescence signal arising from fluorescence was visible to naked eye (**Fig 2B**). The primer set targeting the N gene had an LOD of 25 copies/assay within a 12 min (**Fig 2C**). The primer set targeting the human RNase P gene had an LOD of 44 copies/assay, within a 16 min (**Fig 2D**). Threshold times were compared to RT-qPCR where N gene and RNase P gene were detected in multiplex format (Yang et al., *manuscript in preparation*). For this comparison, Ct values from PCR assay were converted to their corresponding Tt values; RT-LAMP was found to be outperforming over multiplex RT-qPCR in terms of assay rapidity (**Fig 2E**).

### Simple sample preparation of nasal swabs and saliva samples

For a test that can be used at the entrance to a public space to identify carriers who present an environmental risk, sample preparation must be minimal, and any instrumentation involved must be "field-deployable". To be fool-proof, end-point analysis is demanded. Several research groups have also sought low sample preparation workflows, as RNA purification from biological samples is time consuming and timely delivery of test results can be impaired due to limited supplies of sample purification kits [24, 25, 39–42]. To meet these specs, we generated three protocols for SARS-CoV-2 testing.

First, the behavior of the virus itself defines the sampling procedure. False negatives arising from defective sampling are often as problematic as (or more problematic than) false negatives arising from failure of the assay. Fortunately, the life cycle of SARS-CoV-2 appears to allow simple sampling, with mid-turbinate sampling being adequate, as well as saliva sampling [43, 44].

**Fig 3:**
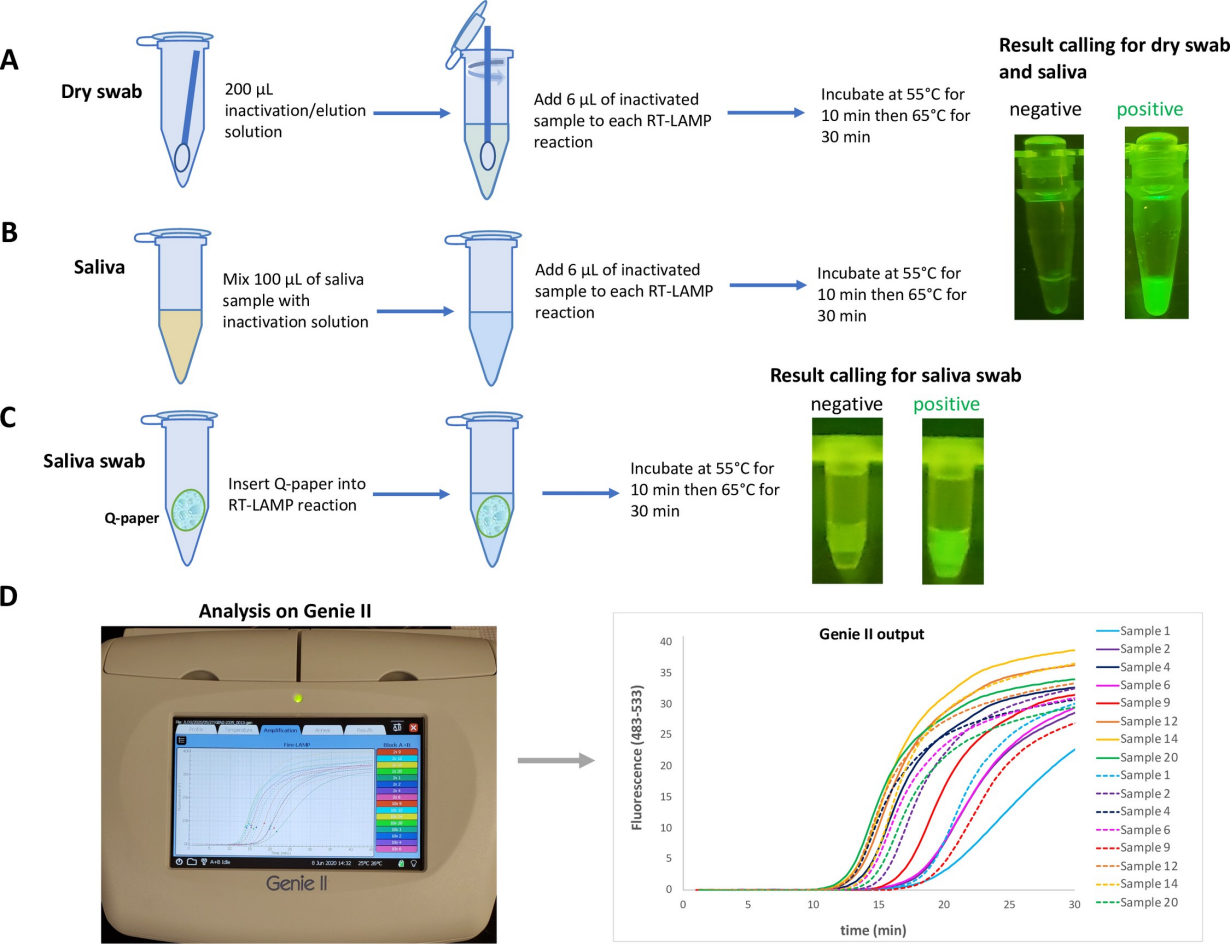
Sampling work-flow and results output. **(A)** Dry nasal swabs were used as sampling method. Swabs were first eluted in a sample preparation buffer and aliquot from that was added into RT-LAMP mixture. End-point results were visualized using blue LED and orange filter. **(B)** Direct saliva was mixed with a sample preparation buffer briefly and aliquot from that was added into RT-LAMP mixture. End-point results were visualized using the same method for nasal swab sampling. **(C)** Q-paper was combined with saliva and Q-paper coated with saliva was directly introduced into RT-LAMP mixture without further manipulation. End-point fluorescent signal was visualized using blue LED and orange filter. Note that the square of Q-paper is observable, but does not compromise the real-time or end-point analysis. **(D)** In addition to end-point visualization, RT-LAMP experiments were also run in real-time using Genie^®^ II (Optigene, UK) which can operate on battery therefore enabling its use in low-resource settings.

Therefore, our first protocol uses dry mid-turbinate or anterior nasal swabbing as a collection method, and relied on the positive control targeting human RNase P to ensure that the collection was adequately aggressive. Post sampling, swabs were eluted in various elution/ inactivation buffers. An aliquot from the elution solution was added directly to the DP-RT-LAMP mixture, and analyzed in real-time using portable Genie^®^ II instrument, available from Optigene and by visualization of end-point fluorescence (**Fig 3A** and **S3A Fig** in **S**1 **File**). Genie^®^ II processes 16 samples simultaneously using the FAM-channel (483–533 nm). The data outputs are similar to those obtained with the more expensive real-time PCR instrument. Genie^®^ II offers positive/negative results with Tt values as good as obtained with the PCR instrument, but at a fraction of the cost and useable in the lobby of a workplace, a courtroom, or a school (**Fig 3D**).

**Fig 4:**
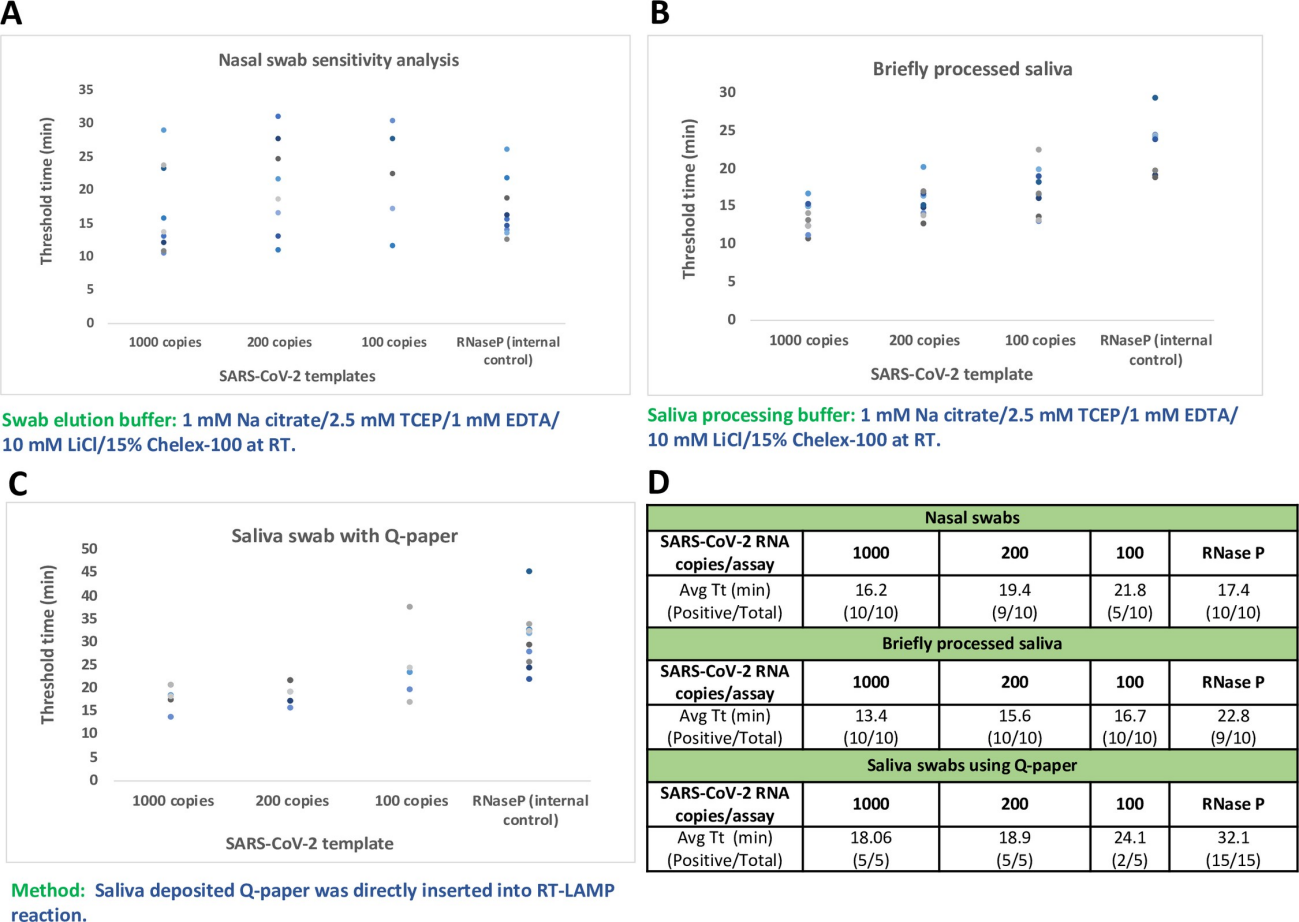
Further evaluation of presently preferred sampling methods and sensitivity analysis with contrived samples using heat-inactivated SARS-CoV-2 template from BEI. **(A)** Varying amounts of RNA was spiked into nasal swab samples from healthy individuals. 200 copies of RNA were detected with consistency and RNase P gene was used as sampling control. **(B)** and **(C)** Varying amounts of RNA was spiked into saliva samples or saliva that was deposited onto Q-paper, respectively. 200 copies of RNA were detected with consistency and RNase P gene was detected successfully. **(D)** Mean Tt values in minutes and numbers of positive results versus total number of samples were displayed in the table.

The sensitivity of the preferred nasal sampling method was determined with a small set of samples using heat-inactivated virus to spike nasal swabs from healthy volunteers. Here, 1000 RNA copies/assay were detected consistently at 100%. Ca. 200 copies/assay were detected with 90% efficiency, and 100 copies of RNA/assay were detected at 50% efficiency. The internal control that targets the RNase P gene was detected at 100%, indicating that the sample collection was sufficiently aggressive (**Fig 4A**).

Additionally, inactivated virus-spiked saliva (with saliva alone as the negative control) was diluted with several inactivation buffers (1:100 ratio of buffer to saliva) and an aliquot of the resulting mixture was added to the DP-RT-LAMP mixture and analyzed similarly (**Fig 3B** and **S3B Fig** in **S**1 **File**). Alternatively, saliva can be placed on "Q-paper", a cellulose filter paper that carries quaternary ammonium groups. Q-paper has been previously used to capture arboviral RNA from single mosquitoes after a drop of ammonia is added to the carcasses [18]. In this work, the Q-paper holding the viral RNA could be added directly to the RT-LAMP mixture without any sample preparation. The fluorescence can be analyzed in real-time or by end-point visualization (**Fig 3C**).

The sensitivities of the assay with two saliva sampling methods were further analyzed using contrived saliva samples of healthy volunteers. Here, 200 copies of RNA/assay could be detected by both methods with 100% detection rate with a small sample size (5 to 10 cases); 100 copies of RNA were detected with 100% efficiency using 100X inactivation solution, and with 40% efficiency using Q-paper for sampling. Additionally, the internal control targeting RNase P gene was detected at 90–100% in both methods (**Fig 4B–4D**).

### Validation of DP-RT-LAMP assay on clinical samples

Previously collected nasopharyngeal (NP) swabs samples that were stored in viral transport medium (VTM) were first eluted at room temperature without a heating step in sample elution buffer used for DP-RT-LAMP assay. SARS-CoV-2 specific primer set and internal control targeting RNase P gene was run in parallel and signal threshold times were determined. LAMP assay results were also compared to a CLIA notified and ICMR (Indian Council for Medical Research) approved PCR assay from GenePath Diagnostics (GPDx CoViDx One PCR). This is a 4-plex real-time RT-qPCR assay targets RdRP, N and E gene of SARS-CoV-2 and uses RNase P gene as sample extraction control. It uses a commercial sample extraction kit to purify and concentrate viral RNA from VTM whereas LAMP method uses minimal sample preparation through simple swab elution. Out of 11 samples tested, LAMP assay results are in 100% agreement with PCR assay with average Tt value of 17 min for SARS-CoV-2 target and 18 min for RNase P targets (**Table 1**).

**Table 1:** Clinical evaluation of DP-RT-LAMP assay and comparable PCR assay.

Sample	LAMP Tt (min)			GPDx CoViDx One PCR Ct				
	CoV2-W3	RNase P	Result	RdRP	N	R	RNase P	Result
1	32.7	24.0	Positive	33.13	35.78	33.26	29.15	Low Positive
2	10.9	18.0	Positive	25.56	27.45	25.25	28.21	Positive
3	41.5	16.2	Positive	30.99	34.24	31.65	25.55	Positive
4	12.4	16.5	Positive	30.85	32.75	31.31	29.81	Positive
5	11.0	16.9	Positive	29.62	31.84	29.72	29.82	Positive
6	16.7	17.7	Positive	31.49	33.73	32.18	31.14	Positive
7	12.7	14.7	Positive	30.6	32.12	30.99	29.25	Positive
8	23.7	15.0	Positive	32.65	35.73	33.31	29.5	Low Positive
9	10.3	15.6	Positive	25.67	27.71	25.75	28.99	Positive
10	13.0	16.7	Positive	31.94	34.87	32.9	27.35	Positive
11	9.0	29.0	Positive	23.77	25.29	23.62	27.51	Positive

LAMP assay: Swab elution in 200 μL buffer. 5 μL input in 25 μL reaction

PCR assay: With RNA extraction (200 μL VTM input, elution volume of 35 μL), 5 μL Input 15 μL reaction.

### Multiplex detection of SARS-CoV-2 and RNase P

**Fig 5:**
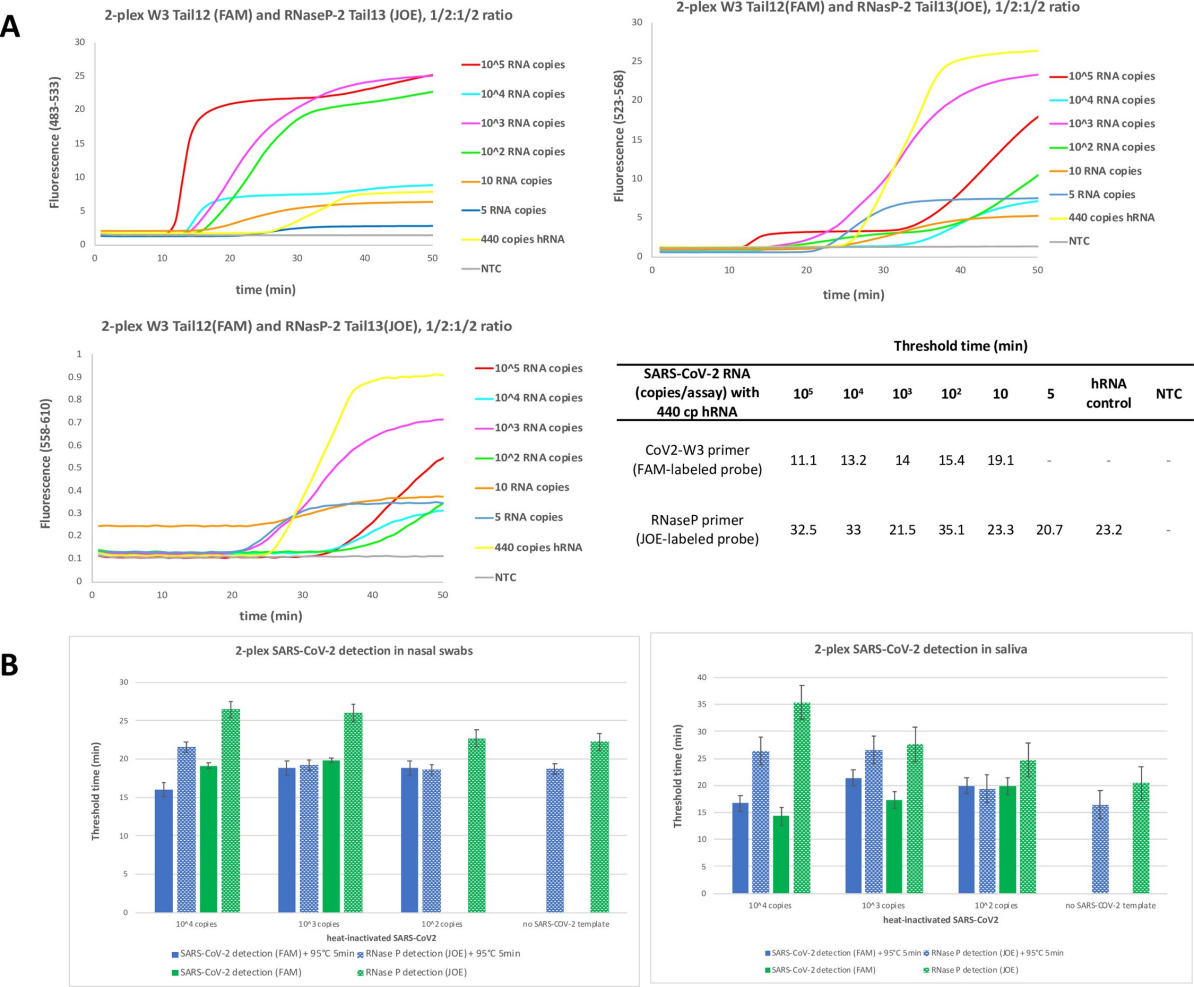
Multiplexed detection of SARS-COV-2 RNA and RNase P (internal control). **(A)** Varying amounts of heat-inactivated SARS-CoV-2 (BEI resources) spiked with human RNA (440 cp). Fluorescence signals from three channels were recorded every 30 seconds using LightCycler^®^ 480. Channel 483–533 is specific for SARS-CoV-2 RNA, channel 523–568 can detect signals from both targets (ladder formation manifests itself), and channel 558–610 is specific for RNase P. Corresponding Tt values were shown on the table. **(B)** 10^4^, 10^3^ and 10^2^ copies of SARS-CoV-2 RNA was spiked into processed nasal swab and saliva samples and analyzed simultaneously.

An assay robust for workplace use must incorporate a signal to indicate that sampling is sufficiently aggressive. Our displaceable probe architecture allows the simultaneous detection of viral RNA and the human RNase P gene in single tube assay. To show this, we spiked varying amounts of viral RNA into human RNA background and 10 copies of SARS-CoV-2 RNA could be detected in the presence human RNA in two-plex format when equal amount of the two LAMP primer sets were present. When viral RNA was present in higher amounts, the signal for RNase P was delayed to 32.5 minutes, instead of appearing between 21–23 min. This is presumed to reflect the two amplification processes competing for some of the LAMP amplification resources. A similar degree of sensitivity for both targets was achieved when viral RNA was ~ 1000 copies/assay (**Fig 5A**).

We then applied the same process to nasal swabs and explored the effect of brief heating step (95°C 5 min) on the assay sensitivity. When no heating step was involved, only 10^4^ and 10^3^ copies of viral RNA could be detected and Tt value of RNase P decreases with the decreasing order of viral RNA input. When samples were heated, more uniform co-amplification of both targets was observed (**Fig 5B**, left panel).

Similar outcomes were observed when saliva samples were used. When there was no heating, 10^4^ and 10^3^ copies of RNA could be detected within 16 min, but signal was delayed to 20 min for 100 copies of RNA. Similar to nasal swabs, RNase P detection was significantly delayed in higher viral load. This is expected of LAMP since the viral amplifications consumed LAMP resources in general (e.g. dNTPs). When saliva samples were heated, more uniform co-amplification of both targets was observed with Tt ranging from 15 to 20 min for CoV-2 and 15 to 25 min for RNase P (**Fig 5B**, right panel).

## Discussion

The SARS-CoV-2 has been especially successful in having itself transmitted worldwide by its ability to cause a wide range of symptoms, ranging from lethality to no recognizable symptoms at all. Further, multiple anecdotal examples show clear transfer of the virus from one symptomatic patient to another symptomatic patient via an individual who displayed no symptoms at all, neither at the time of transmission nor forever after [45]. This makes SARS-CoV-2 virtually unique among pathogens, while presenting an unprecedented challenge to those charged to manage the resulting pandemic. While classical 2003 SARS could be tracked by monitoring symptoms (e.g. fever, which can be remotely tested), SARS-CoV-2 cannot.

This makes the current testing regime totally inadequate. Reports from hospitals and New York City, for example, show the time between sampling, sample transport, and return of sample results ranging from two days to 22 days, with costs per assay generally in excess of $100. Neither this time of delay nor the cost are adequate to determine whether or not the individual being sampled is likely to forward infect other individuals at a workplace, courtroom, jail, or schoolhouse. Indeed, as Thomas Frieden of the CDC pointed out, if the test requires days to resolve an outcome, the test might as well not be taken at all, except for curiosity or for larger epidemiological public health [46].

Compounding this is the fact that the asymptomatic carrier has no particular reason to present himself/herself for testing. The most common way in which such asymptomatic carriers are identified is by broad testing of individuals who have been in contact with asymptomatic patient. However, for serious epidemiological work to be done given the peculiarities of this particular virus, large-scale random testing must be done. Again, the standard procedures involving days to result and $100 per assay are not compatible with this.

It is easy to identify preferred workplace assay; however, it must cost just a few dollars to run. Further, the assay must be run without the need to transport samples or it should survive the transport and storage in amateur hands. Essentially all "home assay" kits are not home assays at all, but rather are home sampling kits.

Further, the results must be returned in minutes, not hours, and certainly not days. Rather, the assay must be usable at the entrance to a workplace, a courtroom, an airport, or schoolyard, and return results in 30 minutes. If the assay is positive, the individual is referred off campus to a reference laboratory. If the assay is negative, to the extent that it does not indicate a risk for forward infection, the individual is allowed to enter the public space.

These specs make certain demands on assay design. First, they must not involve any of the classical sample preparation tests that are used in assays, and reference laboratories. They must be workable by a nonprofessional who need not be licensed, in an environment that must not need CLIA certification. Indeed, they cannot involve a reference laboratory at all.

These demanding specifications may be offset in part by the absence of a need for ultra-high sensitivity. For example, when treating HIV, even 10 virions in a sample indicate that the patient has an infection that demands medical attention. For workplace use, however, an assay need be only as sensitive as necessary to ensure that the individual does not present a forward contamination risk. While the viral load of saliva necessary for that risk is not known, emerging data suggest that this requires hundreds of thousands or millions of viral particles per milliliter for upper respiratory track samples [47].

The assay presented here meets all the requirements for use it in an entrance to a public space, such as a schoolyard, a workplace, or an airport. To ease the need for transport without a chain of refrigeration, assay components were lyophilized and shown to perform similar to their non-lyophilized counterparts. Further, this assay has shown a potential to be used as one-step LAMP where a nasal/saliva swab can be directly eluted into the assay mixture for rapid (<30 min) screening of individuals entering in public spaces (**S5 Fig** and **S4 Table** in **S**1 **File**). It detects virus if it is present at approximately 200 copies per nasal swab assay, representing approximately 8,000 copies of RNA per nasal swab, and 100 copies per saliva assay, representing approximately 20,000 copies of RNA per mL of saliva. This is currently believed to be below the level of mean viral load in upper respiratory specimens [48, 49] and below the level required for a forward infection risk [47, 50].

## Supporting information

S1 File The supplementary information file contains information about primer and probe sequences (S1), initial SARS-CoV-2 templates (S2), optimization of nasal swab sampling (S3), optimization of saliva sampling (S4), lyophilization of DP-RT-LAMP reagents (S5), and one-step LAMP assay using nasal/saliva swabs (S6). (DOCX)
