# Supplementary material for: Ultra-rapid detection of SARS-CoV-2 in public workspace environments
Source: PLoS One. 2021 Feb 24;16(2):e0240524. doi: 10.1371/journal.pone.0240524 (PMC7904170; doi:10.1371/journal.pone.0240524)
Supplement: S1 File — (DOCX) [file pone.0240524.s001.docx]

1. **Primers and displaceable probes**

**Table S1.** LAMP primers and probe sequences used in this study.

| **CoV2_W3 set** | **targeting S gene** |
| --- | --- |
| CoV2-W3-F3 | GAATCTCTCATCGATCTCC |
| CoV2-W3-FIP | AGCAAAGCATAATTGTCACCTTTTTGGCCATGGTACATTTGG |
| CoV2-W3-LF | GGCAATCAAGCCAGCTAT |
| CoV2-W3-LB | TGTGGATCCTGCTGCAA |
| CoV2-W3-BIP | GTCTCAAGGGCTGTTGTTCTTTTTGCTCAGAGTCGTCTTC |
| CoV2-W3-B3 | GACTCCTTTGAGCACTG |
| CoV2-W3-LB-tail12-5IBFQ | /5IABkFQ/GTGTCAAGAGCTCCGAGCCTCGTCGTTCATGCAATAGCGC-TGTGGATCCTGCTGCAA |
| CoV2-tail12-40-Comp3FAM | GCGCTATTGCATGAACGACGAGGCTCGGAGCTCTTGACAC/36-FAM/ |
| **CoV2-v2-4 set** | **targeting N gene** |
| CoV2-v2-4-F3 | ATGACGTTCGTGTTGTT |
| CoV2-v2-4-FIP | CTCCATTCTGGTTACTGCCATTTTTATCAGCGAAATGCACC |
| CoV2-v2-4-LF | TCTGAGGGTCCACCAAAC |
| CoV2-v2-4-LB | ATACTGCGTCTTGGTTCAC |
| CoV2-v2-4-BIP | CGGCCCCAAGGTTTACCTTTTTCCATGTTGAGTGAGAGC |
| CoV2-v2-4-B3 | GGTGTTAATTGGAACGC |
| CoV2-v2-4-LF-tail12-5IBFQ | /5IABkFQ/GTGTCAAGAGCTCCGAGCCTCGTCGTTCATGCAATAGCGC-TCTGAGGGTCCACCAAAC |
| CoV2-tail12-40-Comp3FAM | GCGCTATTGCATGAACGACGAGGCTCGGAGCTCTTGACAC/36-FAM/ |
| **RNaseP-2 set** | **targeting RNase P gene (internal control)** |
| RNaseP-2-F3 | GGAGAGTGAGTTGATCAG |
| RNaseP-2-FIP | ATAGCCCTCCTAGGCTCCTTTTTCCCTCTATCTGCAACTTG |
| RNaseP-2-LF | AGGCTTGCTTACCTCCAG |
| RNaseP-2-LB | CAGAGGCACCTAGGATTGG |
| RNaseP-2-BIP | TGGTGACCTGAACTAGGGTTTTTGTGCTGTGATCTGTCC |
| RNaseP-2-B3 | CTTTCCCTCATCCTTCTC |
| RNaseP-2-LB-tail13-5IBFQ | /5IABkFQ/GCAGCGGACGTCATAGGGACAATATCTTTCTCGCGCGGGA-CAGAGGCACCTAGGATTGG |
| RNaseP-2-tail13-40-Comp3FAM | TCCCGCGCGAGAAAGATATTGTCCCTATGACGTCCGCTGC/36-FAM / |
| RNaseP-2-tail13-40-Comp3JOE | TCCCGCGCGAGAAAGATATTGTCCCTATGACGTCCGCTGC/3Joe_N/ |

Underlined sections are double-stranded portions of displaceable probes. SARS-CoV-2 and RNase P detecting probes were FAM labeled. RNase P probe for multiplexed LAMP was labeled with JOE and Iowa Black FQ was used to quench both fluorophores.

1. **Initial SARS-CoV-2 templates**

*IVT RNA fragment preparation*

Target RNA was generated from synthetic DNA fragment of the viral gene of interest (GenBank: MW075808.1). Synthetic DNA gene fragment was ordered from IDT as gBlocks. An initial PCR introduced the T7 promoter. Next, 150 nM of PCR product was used in T7 RNA transcription reaction (50 µL total volume); the reaction mixture was incubated at 37°C for 16h. DNA templates were removed by digestion with DNase I, the mixture was phenol-CHCl_3_ extracted, and the RNA was recovered by EtOH precipitation. The product RNA was quantified using a Nanodrop UV spectroscopy, and reference materials with known concentrations were prepared in serial dilutions in TE buffer (10 mM Tris pH 7.0, 1 mM EDTA) and aliquots were stored at -80°C.

*Fully synthetic SARS-CoV-2 RNA*

Synthetic SARS-CoV-2 RNA Control was from Twist Bioscience (MT007544.1, 1x10^6^ RNA copies/µL). It was used for initial limit of detection (LOD) studies. Appropriate dilutions were made in 1 mM Na citrate pH 6.5, 0.4 U/µL RNase inhibitor (NEB, Ipswich, MA) and aliquots were stored at -80°C.

*Sensitivity of DP-RT-LAMP assay using IVT RNA and Twist RNA*

Our first experiments sought to measure the sensitivity of a specific RT-LAMP primer set (CoV2-W3) that had been selected from three trial sets that targeted the spike region of the virus genome. Here, RNA target was prepared by transcription of a DNA template (230 nt). Varying concentrations of RNA were used to determine assay sensitivity; assay conditions (65°C, 60 min) followed those established to detect a panel of RNA viruses in mosquitoes [1].

With this target, limits of detection (LODs) were 5 copies/assay, giving a threshold time (Tt, equivalent of Ct) of 22.5 min **(Fig S1A)**. When the synthetic RNA target was replaced by the complete RNA genome (Twist Biosciences, SARS-CoV-2 RNA), the sensitivity dropped to 100 copies/assay with Tt = 25.3 min **(Fig S1B)**.

We then sought conditions to increase the sensitivity of the assay. These included:

(a) adding a second reverse transcriptase (SuperScript IV (SSIV) to the WarmStart reverse transcriptase (WS-RTx, NEB) already present.

(b) changing the reaction buffer,

(c) adding random hexamers (12 µM)

(d) adding excess reverse primer (B3 primer), and

(e) varying the incubation temperature (**Table S2 and Table S3**).

Each reaction mixture was pre-incubated at 55°C for 10 min to ensure formation of sufficient cDNA by the reverse transcriptase. This was then followed by incubation at 65°C.

These modifications improved sensitivity with the full-length RNA genome; LODs improved to 10 copies/assay. This compares favorably with SARS-CoV-2 colorimetric assay from New England Biolabs, which has a reported LOD of 500 copies/assay [2]. However, use of 5X SSIV buffer resulted in fluorescent signal in the absence of target (no template controls, NTCs). This drove the choice of the presently preferred conditions that (i) use the original NEB buffer, (ii) WS-RTx as the only reverse transcriptase, (iii) in the presence of random hexamers, and (iv) or excess B3 primer. These conditions gave no "NTC problem" up to 60 minutes, with an LOD of 10 copies/assay. Refining these conditions further, better Tt values were observed with excess B3 than with random hexamers. Therefore, excess B3 primer was used in further RT-LAMP experiments.

**Fig S1**. **Initial LOD studies using IVT RNA and Twist RNA template.**

**(A)** LAMP assay was run at 65°C for 60 min and LOD was 5 copies/assay with threshold time of 22.5 min. **(B)** LOD was 100 copies/assay with threshold time of 25.3 min for Twist RNA template. NTC= No template control.

**Table S2.** Improvement in the LOD using CoV2-W3 primer set and full-length RNA template (Twist Biosciences) by modifying RT-LAMP conditions

**Table S3.** Variety of RT-LAMP components tested with full length RNA template

(Twist Biosciences)

**Fig S2**. **LOD studies using heat-inactivated SARS-CoV-2 isolate (BEI resources)**

Different enzymes and buffers systems were tested as well as incubation temperature was modified. The determined LOD was 10 copies/assay with a Tt of 16 min using Condition 1.

1. **Optimization of nasal swab sampling**

Having established work-flow parameters, we tested various elution/inactivation buffers with or without a heat step to design the presently preferred protocol. **Fig S3A** summarizes the methods used to process mid-turbinate or nasal anterior swabs. TE (Tris-HCl pH 7.0 and 1 mM EDTA) as an elution buffer gave LODs ≈ 1000 copies/assay, with Tt values of ≈30 min. The procedure of Rabe and Cepko [3] was used, with swabs eluted in buffer containing NaOH, TCEP and EDTA and incubated at 95°C for 5 min, and then spiked with known concentrations of BEI template. These gave LODs as low as 100 copies/assay, with a Tt of 23.5 min. Addition of 15% Chelex-100 to Rabe and Cepko method improved the Tt by 2 min.

Despite its promise, this approach did not give reproducible results when nasal swabs were spiked with inactivated virus prior to the 95°C heating step. A similar problem was observed when same buffer was combined with Chelex-100, in a workflow that incorporated two heating steps, one at 56°C for 15 min and a second at 95°C for 5 min. Dao Thi *et al.* [4] also report similar results when nasal swab elution mixtures were spiked with RNA, and then heated (95°C, 5 min). However, processing of clinical samples using the method developed by Rabe and Cepko [3] with heating at 95°C for 5 min did not cause a decrease in assay sensitivity [5].

Seeking to further simplify sampling work-flow, we modified the elution by replacing NaOH with sodium citrate (pH 6.5) and added TCEP, EDTA, LiCl and Chelex-100. Swabs were eluted at room temperature without any additional heating step. Here, 100 copies of viral RNA were detectable per assay within 18 min, only delayed by 2 min when compared to BEI control template. In addition to real-time analysis, end-point fluorescent images were also visible to human eye at 100 copies/assay (**Fig S3C**).

1. **Optimization of saliva sampling**

Crude saliva was first added to RT-LAMP without any treatment, with a saliva: LAMP reaction mixture ratio of 1:5. As shown in **Fig S3B**, 1000 copies of RNA were detectable only after 40 min. This suggested that crude saliva was not very suitable as a sample on its own.

Suspecting that RNA might be rapidly degraded in saliva, saliva samples spiked with target DNA were tested (**Fig S4**). Here again, the emergence of the signal was substantially delayed, even though the delay was not as large as with the analogous RNA. We then tried nasal elution buffers in more concentrated form. Here, saliva (100 µL) was treated with 100X buffer (0.25 M TCEP, 0.1 M EDTA, 0.1M NaOH or Na citrate, 1 µL) with or without 15% Chelex-100, and with or without a heating step. Multiple runs showed the most reliable results, with no signal in the absence of a template, with inactivation buffer containing TCEP, EDTA, sodium citrate, LiCl and Chelex-100 without a heating step; the LOD was ~ 100 copies/assay. Fluorescent signals obtained from positive samples were clearly differentiable from those arising from samples lacking target (**Fig S3C**).

As an alternative to this saliva sampling method, saliva was absorbed on to Q-paper, which was placed after a brief time (5 min) at room temperature (to simulate how processing might occur in a workplace lobby) and directly added into the RT-LAMP mixture. Analogous to what is seen with mosquito carcasses[6], 100 copies of viral RNA could be detected by simply spotting SARS-CoV-2 RNA onto saliva coated Q-paper from which it was directly amplified by RT-LAMP. Visualization of the positive signals was obtained similarly using blue LED and orange filter combination (**Fig S3D**).

*Q-paper preparation*

Whatman filter paper (1g) was immersed in 1.8% aq. NaOH solution (50 mL) for 10 min. Paper was filtrated and placed in aq. EPTMAC (2,3-epoxypropyl) trimethylammonium chloride) solution (50 mL, ratio of EPTMAC to paper was 0.28) for 24h at RT. Resulting Q-paper was filtrated and neutralized with 1% AcOH (50 mL). Finally, Q-paper was washed three times with 96% Ethanol and dried at 55°C for 1h. Q-paper circles were cut into small rectangles (~ 0.5 x 0.2 cm) for saliva collection.

**Fig S3.** **Optimization of sampling methods and fluorescence visualization with presently preferred methods**. **(A)** Five different methods were evaluated for nasal swab sampling, including (i) TE elution, (ii) the method of Rabe-Cepko *et al*., (iii) a method combining Cepko with Chelex-100, (iv) a method combining Cepko with Chelex-100 with two-step heating, and (v) a process without a heating step. Heat-inactivated SARS-CoV-2 isolate was spiked into nasal swab elutions and each method’s sensitivity was determined. Purified RNA control was included as a reference. **(B)** For saliva sampling, six methods were evaluated: (i) crude saliva without any treatment, (ii) the Cepko method, (iii) Cepko method coupled with Chelex and a heat-step, (iv) Cepko method with Chelex and two-step heating, (v) a process without a heating step, and (vi) deposition of saliva on Q-paper and its direct introduction into RT-LAMP. A purified RNA control was included as a reference. **(C)** End-point visualization of finalized methods: Nasal swab and one of the saliva sampling methods uses buffer solution containing 1 mM Na citrate pH 6.5, 2.5 mM TCEP, 1 mM EDTA, 10 mM LiCl and 15% Chelex-100. LODs for both samplings were determined to be 100 copies/assay **(D)** End-point visualization of saliva deposited on Q-paper and its direct use in DP-RT-LAMP reaction. The LOD was 100 copies/assay using Q-paper.

**Fig S4.** **Analysis of inhibitory effects of saliva.** DNA was used as the spike-in template and Tt values were determined in the absence or presence saliva.

1. **Lyophilization of DP-RT-LAMP reagents**

*Dialysis example for 10 LAMP reactions*

10 µL of Bst 2.0 WarmStart^®^ DNA Polymerase (8U/µL, NEB), 10 µL of WarmStart^®^ RTx Reverse Transcriptase (15 U/µL, NEB), 5 µL of Antarctic Thermolabile UDG (1U/µL, NEB), 5 µL of RNase inhibitor (40U/µL, NEB) was combined with 170 µL of dialysis buffer (10 mM Tris-HCl pH 7.5, 50 mM KCl, 1 mM DTT, 0.1 mM EDTA, 0.1% Triton X-100). 200 µL mixture was dialyzed using ultrafiltration membrane with 10 kDa cut-off limit (13,000 rpm 8 min, Millipore, Billerica, MA) followed by washing twice with 250 µL of dialysis buffer to concentrate resulting glycerol free enzyme mix down to 30 µL. 30 µL of enzyme mix was combined with 25 µL of 10X LAMP primer set, 10 µL of 300 µM B3 primer, 35 µL of dNTP mix (10 mM each of dATP, dCTP, dGTP and 5 mM each of dTTP and dUTP) and 25 µL of 1M D-(+)-trehalose. Combined mixture was then distributed into 8-strip PCR tubes as 12.5 µL aliquots. Samples were frozen by liquid nitrogen and lyophilized for 4-6h. Lyophilized reagents were stored at RT and tested within 7 days.

*Reconstitution of lyophilized DP-RT-LAMP reagents*

Sample (6 µL, nasal swab/saliva or RNA template) was mixed 19 µL of reconstitution buffer (2.5 µL 10X isothermal amplification buffer (NEB), 1.5 µL 100 mM MgSO_4_ and 15 µL of nuclease-free water) and added into lyophilized reagents. RT-LAMP reactions were monitored in real-time using Genie II and fluorescence signal was visualized as described in previous sections.

**Fig S5. Lyophilization work-flow and analysis of dry reagents.**

**(A)** Workflow of lyophilization first involves the removal of glycerol from commercial enzymes. This was done by replacing enzyme storage buffer with its glycerol free version via ultrafiltration. The next step combined 10X primer mix and dNTPs with dialyzed enzymes. The mixture was then frozen (liquid N_2_) and lyophilized for 4-6 hours. **(B)** Lyophilized reagents were activated by supplementing lyophilized reagents with rehydration buffer, and templates containing SARS-CoV-2 RNA or contrived nasal/saliva samples; the DP-RT-LAMP progress was analyzed on Genie II and Tt values were determined. **(C)** End-point fluorescence was visualized using blue LED and orange filter.

1. **One-step LAMP assay using nasal/saliva swabs**

Nasal and saliva swabs collected from healthy individuals were spiked with heat-inactivated virus to simulate a clinical sample. Swabs were then directly eluted in 100 µL of DP-RT-LAMP reactions. Swabs are swirled in reaction tube for 1 time or 3 times. Threshold times to fluorescent signal is shown in Table S4.

**Table S4.** One-step LAMP assay demonstration

|  | Tt Values (min) | | | |
| --- | --- | --- | --- | --- |
|  | **Nasal Swab (1X)** | **Nasal Swab (3X)** | **Saliva Swab (1X)** | **Saliva Swab (3X)** |
| SARS-CoV-2 10^4^ copies | 9.5 | 8.7 | 11.4 | 8.3 |
| Internal Control | 14.8 | 12.6 | 21.6 | 15.7 |

**References**

1. Yaren O, Alto BW, Gangodkar PV, Ranade SR, Patil KN, Bradley KM, et al. Point of sampling detection of Zika virus within a multiplexed kit capable of detecting dengue and chikungunya. BMC Infectious Diseases. 2017;17(1):293. doi: 10.1186/s12879-017-2382-0.

2. Zhang Y, Odiwuor N, Xiong J, Sun L, Nyaruaba RO, Wei H, et al. Rapid Molecular Detection of SARS-CoV-2 (COVID-19) Virus RNA Using Colorimetric LAMP. medRxiv. 2020:2020.02.26.20028373. doi: 10.1101/2020.02.26.20028373.

3. Rabe BA, Cepko C. SARS-CoV-2 Detection Using an Isothermal Amplification Reaction and a Rapid, Inexpensive Protocol for Sample Inactivation and Purification. medRxiv. 2020:2020.04.23.20076877. doi: 10.1101/2020.04.23.20076877.

4. Dao Thi VL, Herbst K, Boerner K, Meurer M, Kremer LPM, Kirrmaier D, et al. Screening for SARS-CoV-2 infections with colorimetric RT-LAMP and LAMP sequencing. medRxiv. 2020:2020.05.05.20092288. doi: 10.1101/2020.05.05.20092288.

5. Anahtar MN, McGrath GEG, Rabe BA, Tanner NA, White BA, Lennerz JKM, et al. Clinical assessment and validation of a rapid and sensitive SARS-CoV-2 test using reverse-transcription loop-mediated isothermal amplification. medRxiv. 2020:2020.05.12.20095638. doi: 10.1101/2020.05.12.20095638.

6. Yaren O, Alto BW, Bradley KM, Moussatche P, Glushakova L, Benner SA. Multiplexed Isothermal Amplification Based Diagnostic Platform to Detect Zika, Chikungunya, and Dengue 1. JoVE. 2018;(133):e57051. doi: doi:10.3791/57051.
